# Bioresorbable plate fracture after cranioplasty caused by head injury: a pediatric case

**DOI:** 10.1186/s12245-021-00401-5

**Published:** 2021-12-20

**Authors:** Kohei Igarashi, Atsushi Kuge, Hiroshi Homma, Tetsu Yamaki, Rei Kondo, Shinjiro Saito, Yukihiko Sonoda

**Affiliations:** 1 Department of Neurosurgery, Yamagata City Hospital Saiseikan, Nanokamachi 1-3-26, Yamagata, 990-8533 Japan; 2 Department of Emergency Medicine, Yamagata City Hospital Saiseikan, Nanokamachi 1-3-26, Yamagata, 990-8533 Japan; 3 Department of Neurosurgery, Yamagata University, Faculty of Medicine, Iidanishi 2-2-2, Yamagata, 990-2331 Japan

**Keywords:** Bioresorbable plate, Cranioplasty, Pediatric, Head injury

## Abstract

**Background:**

Recently, bone fixation materials have been developed as surgical materials. Bioabsorbable materials offer several advantages over other materials and are widely used. We report a rare case of the fracture of bioresorbable plates caused by head injury and describe some considerations.

**Case description:**

A 6-year-old boy suffered from consciousness disturbance. He was admitted to our hospital and diagnosed with left frontal subcortical hemorrhage due to ruptured arteriovenous malformation (AVM). He received the surgery of removal of the AVM with decompressive craniectomy. He was discharged without any neurologic deficit and underwent the cranioplasty 4 months after the initial surgery. Two months after the last treatment, he was fallen and hit his left frontal head. The next day, he noticed an abnormal bulge in the injured area. We diagnosed the bulging as cerebrospinal fluid leakage because of the dural tear. The repairment of dural tear was performed. We found that two bioresorbable plates used by cranioplasty were both cracked, and the dura mater beneath them was torn. We repaired the damaged dura with an artificial dura mater. After surgery, cerebrospinal fluid leakage did not occur.

**Conclusion:**

It has been reported that the durability of bioresorbable plates is no less than that of titanium plates. We experienced a relatively rare case in which bioabsorbable plate used for bone fixation was damaged due to head trauma. After craniotomy or cranioplasty using bioresorbable plates, special attention should be paid to head trauma that involves bone flap sinking force and side bending stress.

## Introduction

Various bone fixation materials have been developed [9, 19]. Bioabsorbable plates are slightly less durable than titanium plates but are comparable to titanium plates in osteosynthesis [1–3, 8, 13, 22]. Bioresorbable osteofixation materials offer several advantages over titanium fixation, including the absence of the need to remove the implants after osseous healing, radiolucency, and decreased pain. Considering these advantages, the use of bioresorbable plates is increasing, especially in pediatric cases [4]. We report a very rare case of the fracture of bioresorbable plates caused by head injury and describe some considerations.

## Case report

**Fig. 1:**
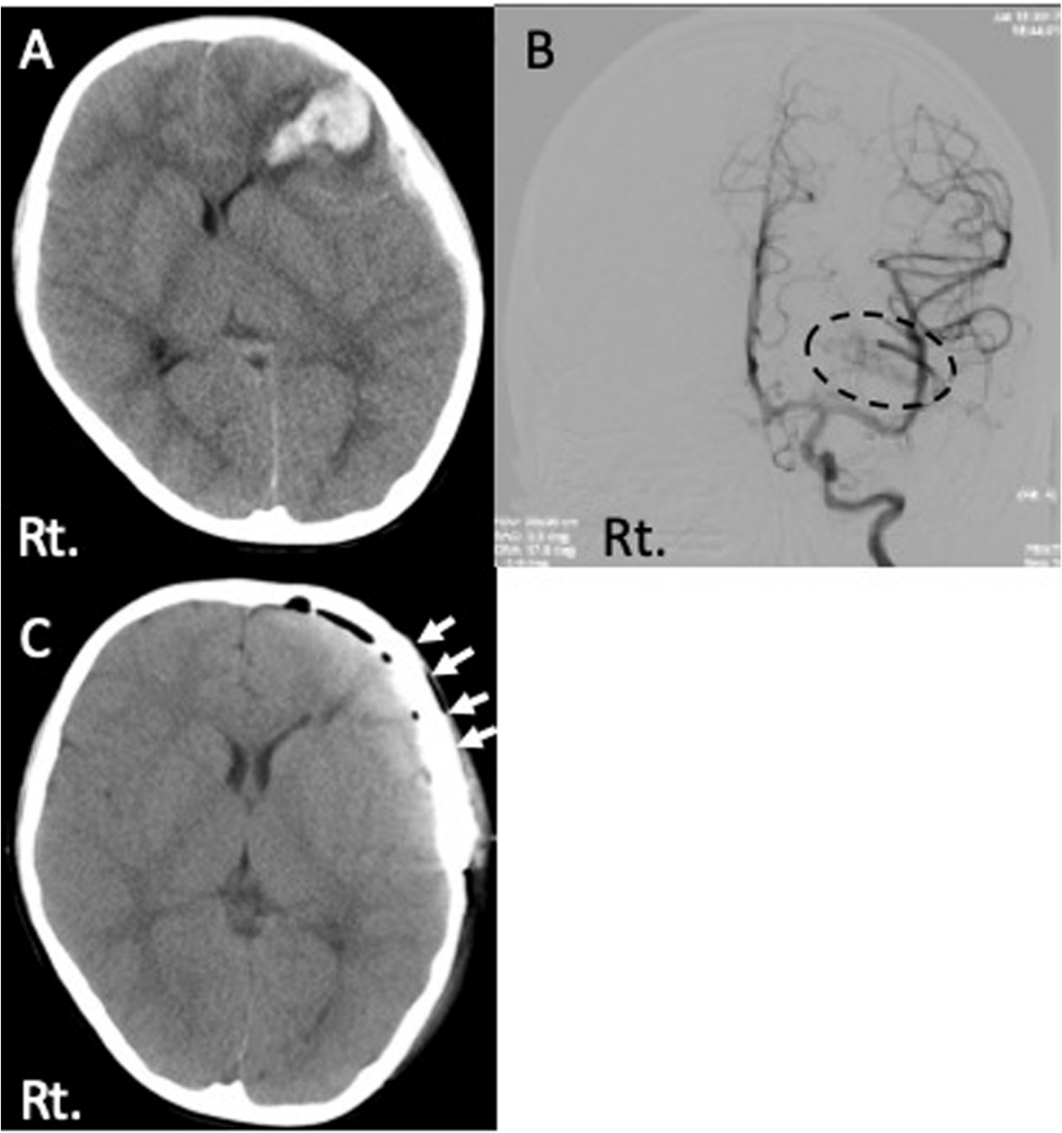
Radiological examinations at first treatment. **a** Head CT showed left frontal subcortical hematoma. **b** Cerebral angiography of the left internal carotid artery showed AVM adjacent to the hematoma (dotted ring). **c** CT image after cranioplasty

A 5-year and 11-month-old boy presented with left frontal subcortical hematoma due to ruptured AVM and underwent the removal of AVM with the decompressive craniectomy [Fig. 1a, b]. Four months after the initial surgery, cranioplasty was performed using microporous hydroxyapatite (APACERAM®). We used two bioresorbable plates and eight screws (Lactosorb®) at the frontal region and one titanium plate at the temporal region to fix the artificial bone flap [Fig. 1c]. He was discharged without any neurological deficit after initial treatment.

Nine weeks after the cranioplasty, he hit his left forehead on a refrigerator. The next day, he noticed an abnormal bulge in the injured area. He took a medical check at our department because the bulging area was increased, and there were no signs of recovery. At the time of our check, his consciousness was clear, and he had no other neurologic deficit. The bulging area was located where we performed cranioplasty at the initial treatment. The bulging area was soft, and there were no signs of inflammation.

**Fig. 2:**
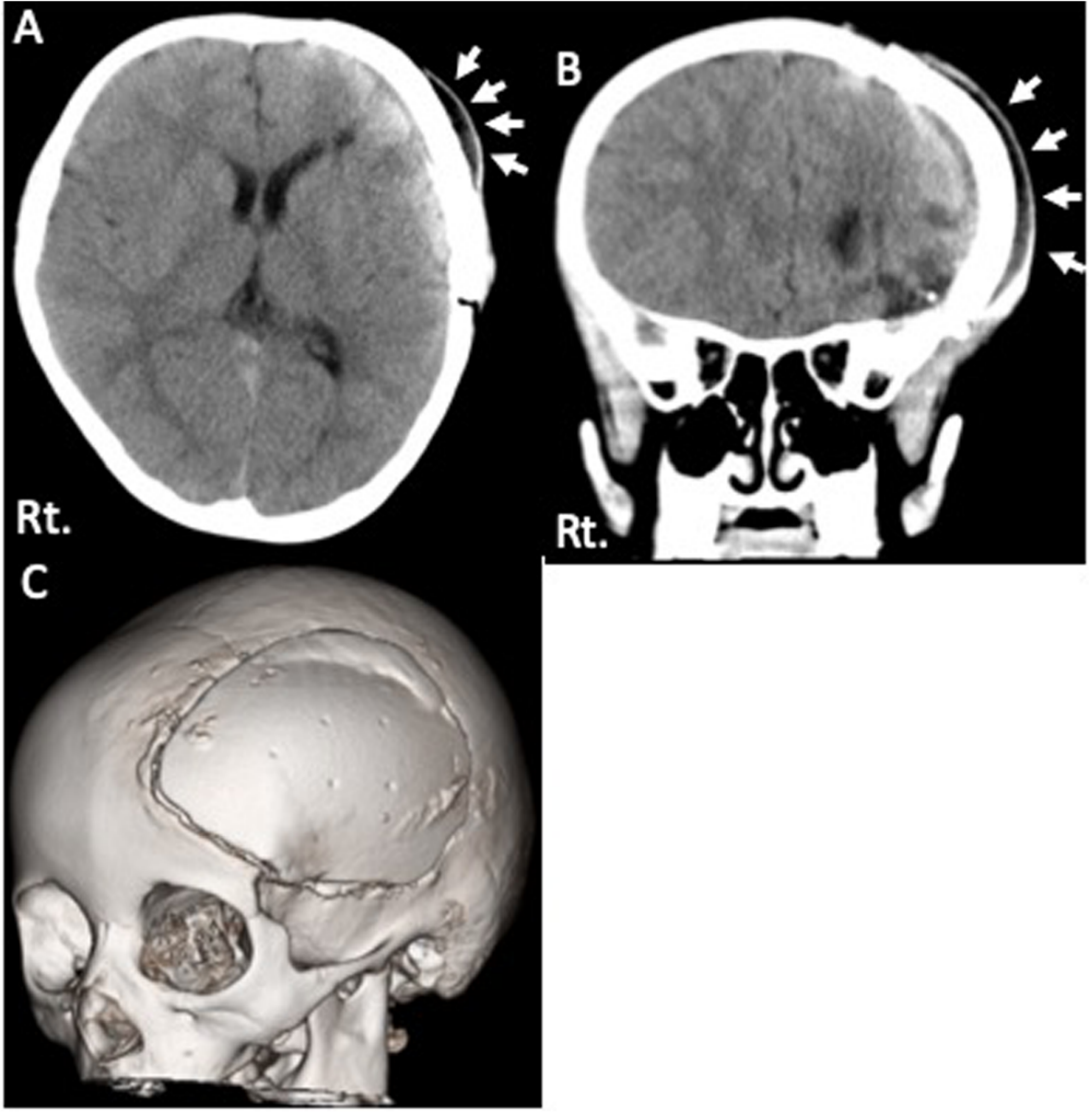
Radiological examination after head injury. **a**, **b** Head CT showed subcutaneous fluid collection (white arrows). **c** Three-dimensional image showed the relationship between the artificial bone flap and skull. No obvious deviation of the bone flap

Head computed tomography (head CT) showed the fluid collection under the scalp and epidural space [Fig. 2]. The CT Hounsfield number of this lesion was low; this finding was suggesting the collection of cerebrospinal fluid rather than that of bleeding. We speculated that the cerebrospinal fluid leakage had occurred due to a dural laceration at the previous surgical site. We decided to surgically repair this lesion based on our speculation.

### Operative findings and follow-up

**Fig. 3:**
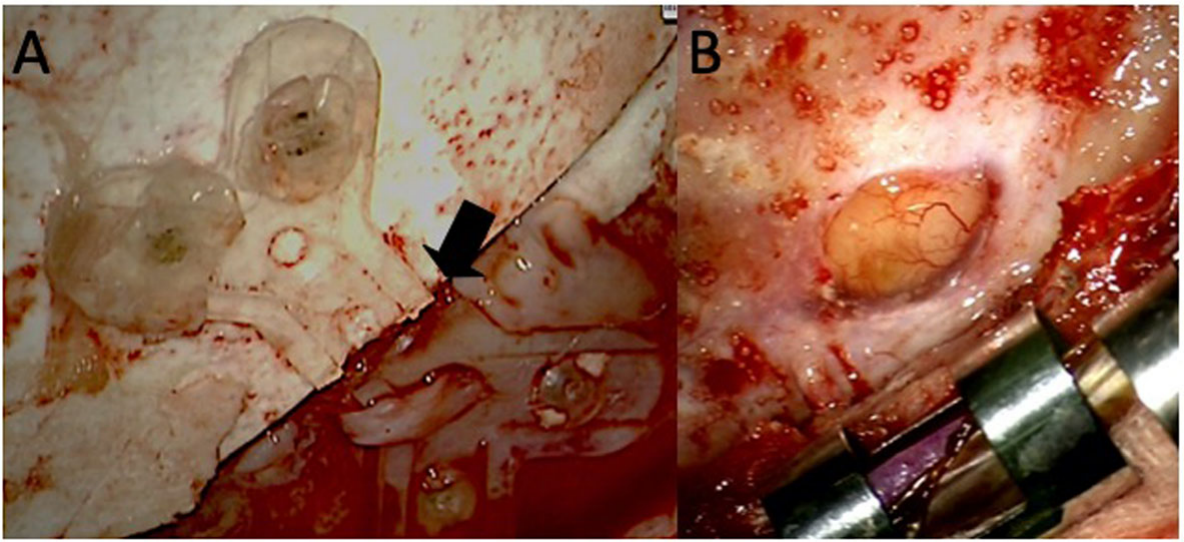
Intraoperative photos. **a** Bioresorbable plates were fractured in the middle (black arrow). **b** The dural tear at the edge of the bone flap and the cerebrospinal fluid was leaking at this point

The previous skin incision was made, and the skin flap was flipped. Though we found no fracture of the artificial bone flap, two bioresorbable plates we used previously were both fractured in the middle [Fig. 3a]. After taking off the bone flap, there was a dural tear at the point of the bone edge, and the cerebrospinal fluid was leaking. [Fig. 3b]. We repaired the injured dura with polyglycolic acid (Dura wave®) with fibrin glue. We fixed the bone flap previously used again by a titanium plate. After the repairment, the bulging area vanished, and he was discharged without any adverse effects. We have followed him for 3 years, no troubles have been seen of his skull.

## Discussion

When we fixed the bone flap with metal wire or suture thread such as silk or nylon, the fixation was not enough and some problems often happened [3, 9]. The osteofixation of titanium plates has been developed. Titanium plates are able to fix a bone flap easily but also rigidly. Thus, titanium plates are now widely used at craniotomy. However, adverse events associated with titanium plates are reported especially for pediatric cases implanted for a long period. Especially for periatric patients, these are very important points that adverse outcomes include deviation of the plates, inhibition of cranial bone growth, aberrance into the brain, scalp thinning, and plate exposure [6, 7, 9, 14, 20].

Recently, various bioresorbable osteofixation implant materials have been developed. It has been reported that polyhydroxyl acids, poly-d-lactic acid, polyglycolic acid, etc. are the materials. The first use of bioresorbable implants to animals was published in 1966 by Kulkarni et al. [11]. Several reports described that adverse effects were not different between bioresorbable and titanium materials, so bioresorbable materials are not inferior to titanium materials [1–3, 8, 12, 13, 22]. Bioresorbable materials are inferior to titanium in terms of fixing strength, and biocompatibility is not inferior to titanium.

Bioresorbable materials are absorbed in about a year, eliminating the need to remove the implant after osseous healing, and bioabsorbable materials benefit from reduced tactile sensation, pain, and radiolucency. (Table 1) [4, 9, 19]. Because of those advantages described above, the use of them for pediatric cases has been increasing.

**Table 1:**
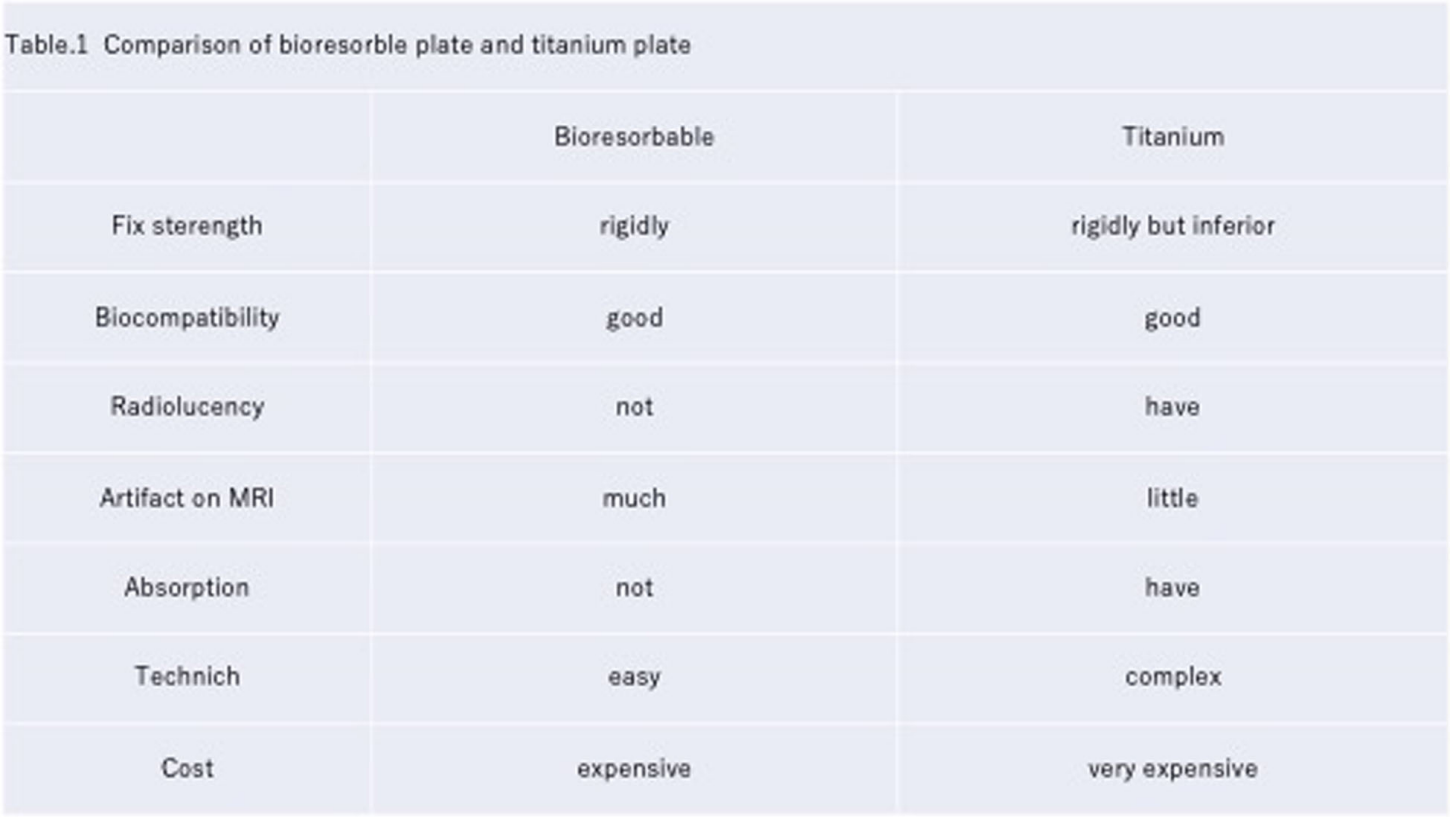
Comparison of the bioresorbable plate and titanium plate comparison of the bioresorbable plate and titanium plate

Our case showed the fracture of bioresorbable plates after head injury. Lactosorb® which we used in this case is made from a copolymer of 82% poly-l-lactic acid and 18% polyglycolic acid. In vitro exposure, it is reported that it retains about 70% of the initial shear strength after 8 weeks. Thus, it is considered that Lactosorb® fix strength has retained until natural osseous healing [11, 17, 21]. The time of resorption of the copolymer plate is about a year [12, 14]. The screws made of the same material have about 80% of the initial shear strength at 4 weeks and keep the strength after that [18]. It is also reported that bioresorbable plate has similarly bending and tensile stiffness as titanium plate but showed low side bending stiffness of the edges of the bioabsorbable plate [15].

APACERAM® which we used as a bone flap at cranioplasty is made from microporous hydroxyapatite. Newly formed bone was detected on the surface of the material and in the macropores near the surface 1 week after transplantation, and it was reported that the compressive strength of 10 MPa is maintained after 5 weeks [21].

This case was injured 8 weeks after the cranioplasty and was thought to have been about 70% of the initial shear strength of the plate at that time. In this case, it was probable that the bone flap sank due to head injury, and the bioabsorbable plate was subjected to lateral bending stress, resulting in the division at the central part. As a result, the dura mater was injured by the edge of the bone flap and cerebrospinal fluid leakage occurred and made the skin bulge.

By the time the bioabsorbable plate was absorbed, it is thought that bone healing and adhesion to the peripheral bone would progress and shear strength would be maintained [10, 16]. In this case, the bioabsorbable plate was damaged by a head injury before such a condition occurred. This case indicated that it is necessary to pay much attention to early head injury after cranioplasty with bioresorbable plates.

## Conclusion

We reported the case of the fracture of bioresorbable plates caused by head injury. After craniotomy or cranioplasty using bioresorbable plates, it is necessary to pay attention to the head injury, especially for bone flap sinking and side bending stress.

## Abbreviation

AVM: Arteriovenous malformation
CT: Computed tomography
